# Therapeutic potential and molecular mechanisms of Yi-Yi-Fu-Zi-Bai-Jiang-San in the treatment of colorectal cancer

**DOI:** 10.3389/fphar.2026.1813778

**Published:** 2026-08-14

**Authors:** Jinxiao Li, Keke Jiang, Shuhan Wang, Yuju Cai, Luorui Shang, Fangyuan Zhou, Yuqing Wang, Jianghua Huang, Shan Hu, Qian Yan, Shenglan Yang

**Affiliations:** 1 Department of Clinical Nutrition, Union Hospital, Tongji Medical College, Huazhong University of Science and Technology, Wuhan, China; 2 State Key Laboratory of Traditional Chinese Medicine Syndrome, Guangzhou, China; 3 The First Affiliated Hospital of Guangzhou University of Chinese Medicine, Guangzhou, China

**Keywords:** colorectal cancer, gut microbiota, mechanism, research progress, YYFZBJS

## Abstract

Colorectal cancer (CRC) is one of the most common gastrointestinal tumors with elevated prevalence and high mortality rates, posing a serious threat to human health. As a commonly used classic prescription in the clinical treatment of CRC, Yi-Yi-Fu-Zi-Bai-Jiang-San (YYFZBJS), which is composed of *Coix lacryma-jobi L., Aconitum carmichaelii Debeaux,* and *Patrinia scabiosifolia Fisch. ex Trev.,* exerts anti-CRC effects by regulating inflammation-cancer transformation, alleviating oxidative stress, restoring gut microbiota balance, inhibiting CRC cell proliferation, promoting apoptosis, regulating autophagy, suppressing tumor angiogenesis. It demonstrates multi-targeted anti-CRC potential and holds promising clinical value as a chemotherapy adjuvant. However, the molecular mechanisms underlying its multi-target anti-CRC effects remain incompletely elucidated and require comprehensive synthesis. This review summarizes the research progress of YYFZBJS in recent years in the field of CRC. It will provide theoretical basis and ideas for further investigation of the role of YYFZBJS in anti-CRC, with the ultimate goal of benefiting the clinical practice.

## Graphical Abstract

**Figure:**
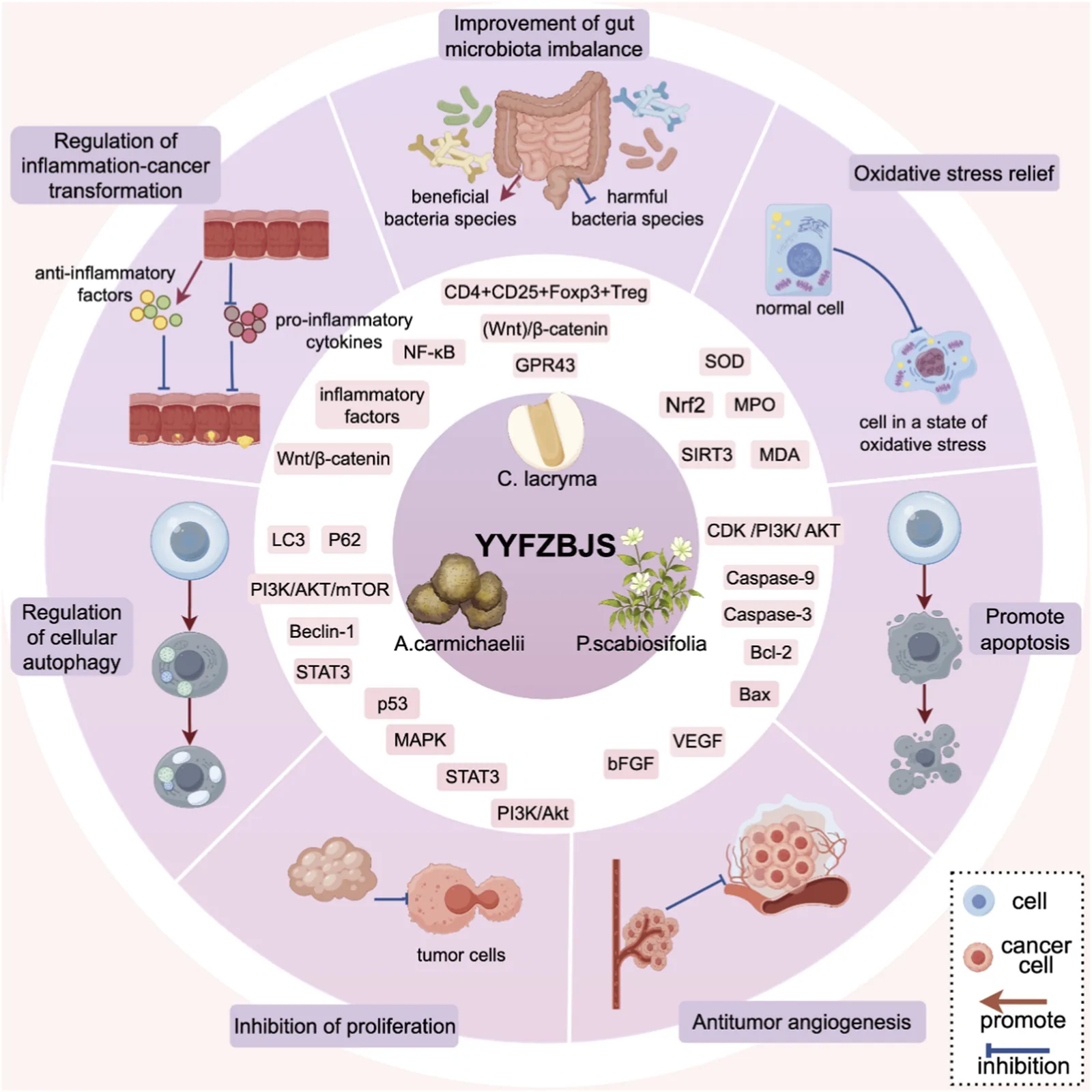

## 1 Introduction

Colorectal cancer (CRC) is one of the most common gastrointestinal tumors with high prevalence and high mortality rate. The incidence of CRC has increased globally in recent years, accounting for about 10% of all cancers and becoming the second most common cause of cancer deaths (Bray et al., 2024; Siegel et al., 2026). National estimates for China in 2022 indicate that CRC ranked second among all cancers by number of new cases (approximately 517,100) and fourth by number of cancer deaths (approximately 240,000) (Han B. et al., 2024). Colorectal cancer (CRC) represents a substantial global cancer burden and remains an important contributor to cancer-related morbidity and mortality worldwide.

Cancer therapeutics have undergone successive paradigm shifts: from cytotoxic chemotherapy in the second half of the 20th century, to molecularly targeted agents in the early 2000s, and more recently to immune checkpoint inhibition and adoptive cell therapy. Each generation has expanded the therapeutic toolkit, yet CRC continues to illustrate the limits of any single-modality strategy—cumulative drug resistance, microsatellite-stable tumours that respond poorly to immune checkpoint blockade, and considerable inter-individual variation in toxicity (Wang X. et al., 2025; Xie et al., 2020; Simoes et al., 2020; Sonkin et al., 2024). Against this backdrop, multi-component traditional Chinese medicine (TCM) formulae such as YYFZBJS have re-emerged as candidates for adjuvant and chemo-protective use, because their pharmacology is inherently network-based and may complement, rather than compete with, the precision-oriented logic of modern oncology (Zou et al., 2024). Currently, the treatment of CRC mainly includes surgery, chemotherapy, radiotherapy, targeted therapy, and immunotherapy (Fan et al., 2021). However, existing treatment options have limitations such as chemotherapy resistance and drug side effects, which may potentially be alleviated by Chinese medicine. Emerging clinical and preclinical evidence indicates that Chinese herbal medicine, used as adjuvant therapy alongside conventional regimens, may attenuate chemotherapy-related toxicity, improve quality-of-life scores and, in selected populations, prolong progression-free survival. (Tian et al., 2024; Chen X. et al., 2023). As a classic prescription for intestinal disorders, YYFZBJS was first published in *the Synopsis of Golden Chamber*: “intestinal abscess is treated with YYFZBJS”. Numerous studies have demonstrated that this formula effectively enhances therapeutic efficacy while reducing toxicity, and inhibits tumor recurrence and metastasis in the treatment of CRC (Lin et al., 2023; Lv et al., 2022; Nie, 2020). In this review, we will summarize the current research status of YYFZBJS in the prevention and treatment of CRC, and provide a reference for the in-depth study of the mechanism of action of YYFZBJS in the prevention and treatment of CRC and its wide application in clinical practice.

## 2 Traditional Chinese medicine (TCM) theory and CRC pathogenesis

According to Chinese medicine theory, CRC is generally regarded as a disease of deficiency in origin and excess in superficiality, in which the weights of deficiency and excess shift across stages.

In the early and intermediate stages, excess patterns predominate. Dietary irregularities—excessive consumption of fatty, sweet, pungent, dry or contaminated foods—coupled with invasion of damp-heat and toxin impair the spleen-stomach transportation and transformation function; phlegm, dampness and blood stasis become intertwined with damp-heat–toxin and lodge in the large intestine, consolidating into a mass. In modern terms, damp and heat correspond to endotoxemia triggered by gut microbiota dysbiosis (decreased *Bifidobacterium*, increased *Enterobacteriaceae*), and toxin corresponds to tumor angiogenesis induced by NF-κB pathway activation (Jiang et al., 2025); sustained release of pro-inflammatory cytokines (e.g., TNF-α, IL-6) causes DNA damage and accelerates adenoma-to-carcinoma progression (Lin et al., 2021; Li and Hua, 2017). Phlegm-dampness coagulation parallels tumor stromal fibrosis and aberrant extracellular matrix (ECM) deposition (Zhang et al., 2026), and its interplay with blood stasis phenotypically resembles cancer-associated fibroblasts (CAFs), which facilitate invasion and metastasis through pro-fibrotic factor secretion and ECM remodeling (Sun et al., 2016).

With progression to advanced disease (typically stage III–IV) does vital qi deficiency become a defining feature. Chronic damp-heat, phlegm and blood stasis, together with the consumptive effect of the tumor, gradually deplete spleen qi and may evolve into qi–blood or spleen–kidney yang deficiency; the mass further blocks the flow of qi and blood in the spleen and stomach, progressively leading to intestinal obstruction (Li et al., 2017). This late-stage spleen deficiency corresponds to an immunosuppressive intestinal microenvironment that disrupts immune cells, impairs intestinal barrier function and aggravates dysbiosis (Shen et al., 2024; Ning et al., 2024), which together promote endogenous carcinogenesis (Yan et al., 2022).

The therapeutic principle is therefore stage-adapted: elimination of pathogenic factors predominates in the early–intermediate stages, whereas both reinforcement of vital qi and elimination of pathogens are required in advanced disease. Based on this TCM pathogenic theory, YYFZBJS, with its “discharging pus for eliminating carbuncles and warming yang for resolving masses” efficacy. YYFZBJS was originally a common formula for treating intestinal abscess. It has the efficacy of warming Yang and fortifying the spleen, clearing heat and resolving dampness, draining pus and removing toxins, breaking up blood stasis and dispersing knots, and has been clinically used for a variety of diseases with favorable results (Chai et al., 2020).

## 3 Pharmacological evidence for active compounds of three constituent herbs in YYFZBJS

### 3.1 C. lacryma

*Coix lacryma*, the dried mature seed kernels of the plant, was first documented in *the Shen-nong’s Classic of the Materia Medica* and is recognized for its potent antitumor activities, including the inhibition of angiogenesis (Jiang et al., 2000), cell proliferation (Chang et al., 2003), and cyclooxygenase (COX) activity (Hung and Chang, 2003; Woo et al., 2007).Modern pharmacological studies have identified various chemical components from *C. lacryma*, including esters, starch, fatty acids, polysaccharides, proteins, phenolic acids, sterols, flavonoids, lactam, triterpenoids, alkaloids, adenosine, etc (Han S. et al., 2024). Among these, coixenolide (diterpenoid ester, ≥0.2 mg/g), linoleic acid (unsaturated fatty acid, ∼15% of total fatty acids), coixan (heteropolysaccharide, 50–100 kDa), and coixol (lactam, 0.08–0.12 mg/g) are the primary antitumor-active compounds. Coixenolide inhibits TNF-α-mediated epithelial-mesenchymal transition (EMT) in CRC cells, while linoleic acid reduces immune damage caused by 5-FU chemotherapy and Coixol inhibits the NF-κB and MAPK pathways (Lee et al., 2023; Shi et al., 2018; Wang H. et al., 2024; Wu and Yuan, 2010). Coixan, a *C. lacryma* polysaccharide composed of glucose, mannose, and arabinose in a molar ratio of 5:2:1, is another important antitumor constituent that induces apoptosis in lung cancer cells by upregulating Caspase-3 and Caspase-9 and may also, together with CSP1/CSP2, inhibit A549 cell migration and invasion by downregulating S100A4 and interfering with S100A4–NMIIA binding (Lu et al., 2013; Luo et al., 2017).

**FIGURE 1:**
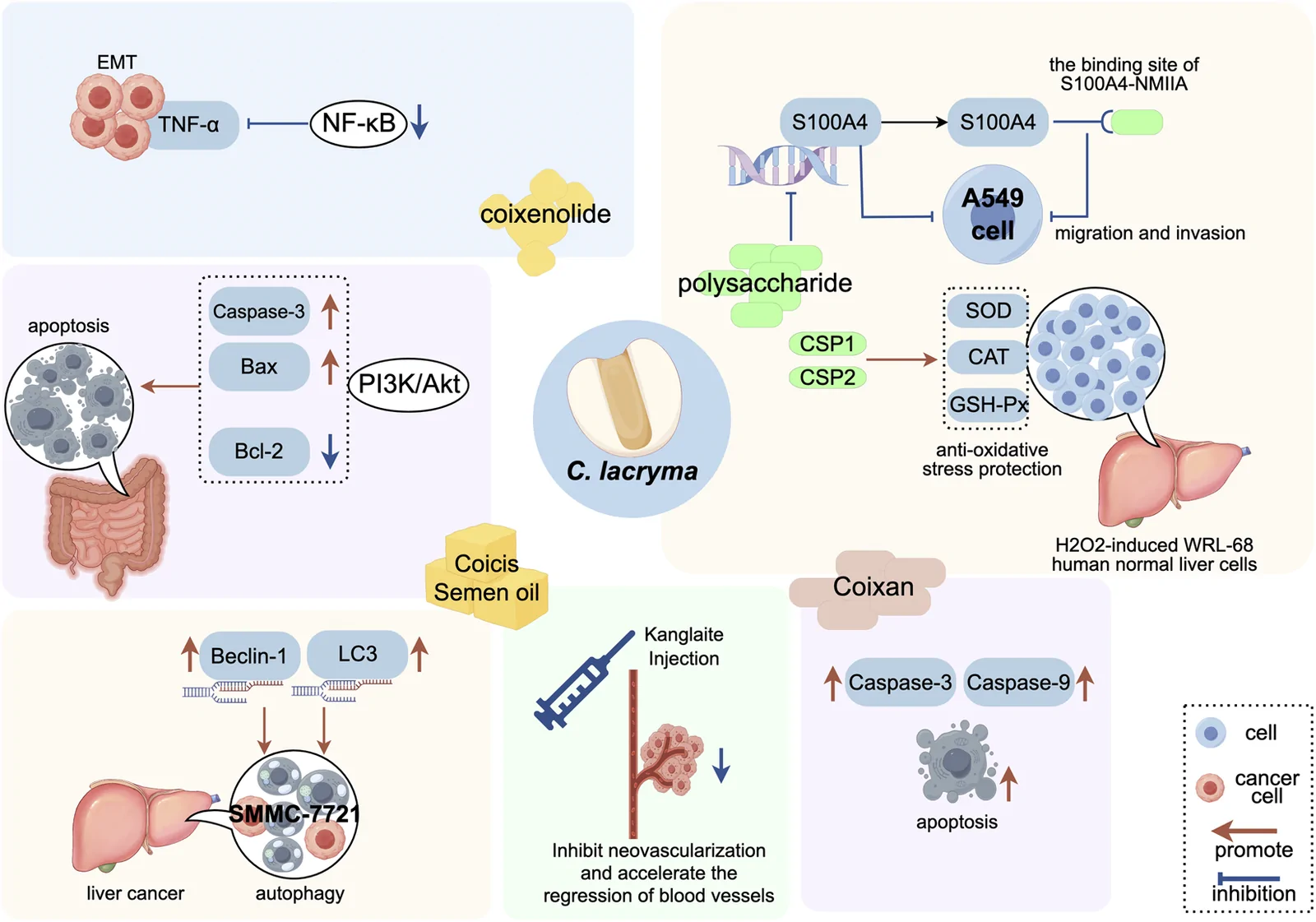
Pharmacological evidence for *C. lacryma*.

The clinical translation of these mechanisms is best exemplified by Kanglaite Injection, a dual-phase antitumor agent that inhibits malignant growth while bolstering systemic immunity (Liu J. et al., 2019). Its primary constituent is *C. lacryma* oil extracted via supercritical CO_2_ extraction with a yield ≥8%; its main components are plantago glycoside and linoleic acid (Zheng, 2024). Studies have demonstrated that *C. lacryma* oil can affect colon carcinogenesis by regulating the PI3K/Akt signaling pathway (Shao et al., 2025), activating Caspase-3, up-regulating Bax, and down-regulating Bcl-2 to induce apoptosis (Ni et al., 2021) (Pharmacological evidence for *C. lacryma* is shown in Figure 1).

### 3.2 A. carmichaelii

*A. carmichaelii*, first recorded in *the Divine Husbandman’s Classic of the Materia Medica*, is a processed product of the root of *Aconitum carmichaelii Debeaux* from the buttercup plant, family *Ranunculaceae*.

Earlier studies have shown that aconite alkaloids possess significant antitumor efficacy (Gao et al., 2022; Wu et al., 2014). Among these compounds, aconitine alkaloids are the most potent active constituents. The type of alkaloids is dominated by C19 diterpenoid alkaloids, which represent one of the plant components with the highest number of compounds and the most toxic compounds identified to date (Tang et al., 2017). In addition, C18 diterpenoid alkaloids from the processed root have been associated with p38 MAPK activation and increased expression of DR5 and TNF-R1, implying that death receptor-related apoptotic signaling may participate in their antitumor effects. However, these findings are mainly based on molecular marker changes and do not by themselves establish pathway causality. Rescue experiments using p38 MAPK inhibitors, caspase inhibitors, or genetic perturbation would be required to determine whether these pathways are necessary for the observed cytotoxic or apoptotic phenotype (Zhang et al., 2022). In human lung cancer A549 cells, aconite C19 diterpenoid alkaloids have been reported to increase the expression of Beclin-1, LC3-II, Bax, and Caspase-3, while decreasing p62 and Bcl-2, suggesting the possible involvement of autophagy- and apoptosis-related processes (Zhang et al., 2022). *In vivo*, the extract has been reported to inhibit the growth of transplanted liver cancer H22 tumors. When combined with cyclophosphamide, aconite extract promoted the expression of TNF-α and Caspase-3, inhibited NF-κB expression, and was associated with enhanced tumor cell apoptosis (Ren and Zeng, 2008). Aconite polysaccharides with a molecular weight 20–30 kDa can alleviate cyclophosphamide-induced immunosuppression by increasing spleen and thymus indices, promoting T- and B-cell proliferation, elevating serum IFN-γ and Th1 cytokine secretion, and enhancing macrophage phagocytosis and NK-cell activity (Fu et al., 2018). Clinically, *A. carmichaelii* and its formulations have also been reported to relieve cancer-related fatigue and pain, suppress tumor progression, and mitigate post-chemotherapy myelosuppression (Lin et al., 2023; Li L. et al., 2020; Li and Zhang, 2019).

It should be emphasised that most antitumor mechanisms attributed to *A. carmichaelii* alkaloids derive from expression-level read-outs (Western blot of Bax, Bcl-2, Caspase-3, LC3-II, p62) in a limited number of cell lines, without parallel pharmacological or genetic rescue. Whether the observed apoptotic or autophagic phenotype is causally driven by p38 MAPK activation, NF-κB suppression or caspase engagement therefore remains correlative, and should be confirmed in CRC models using selective MAPK inhibitors, pan-caspase blockade or pathway-specific knockdown before these axes are accepted as the dominant *in vivo* mechanism.

**FIGURE 2:**
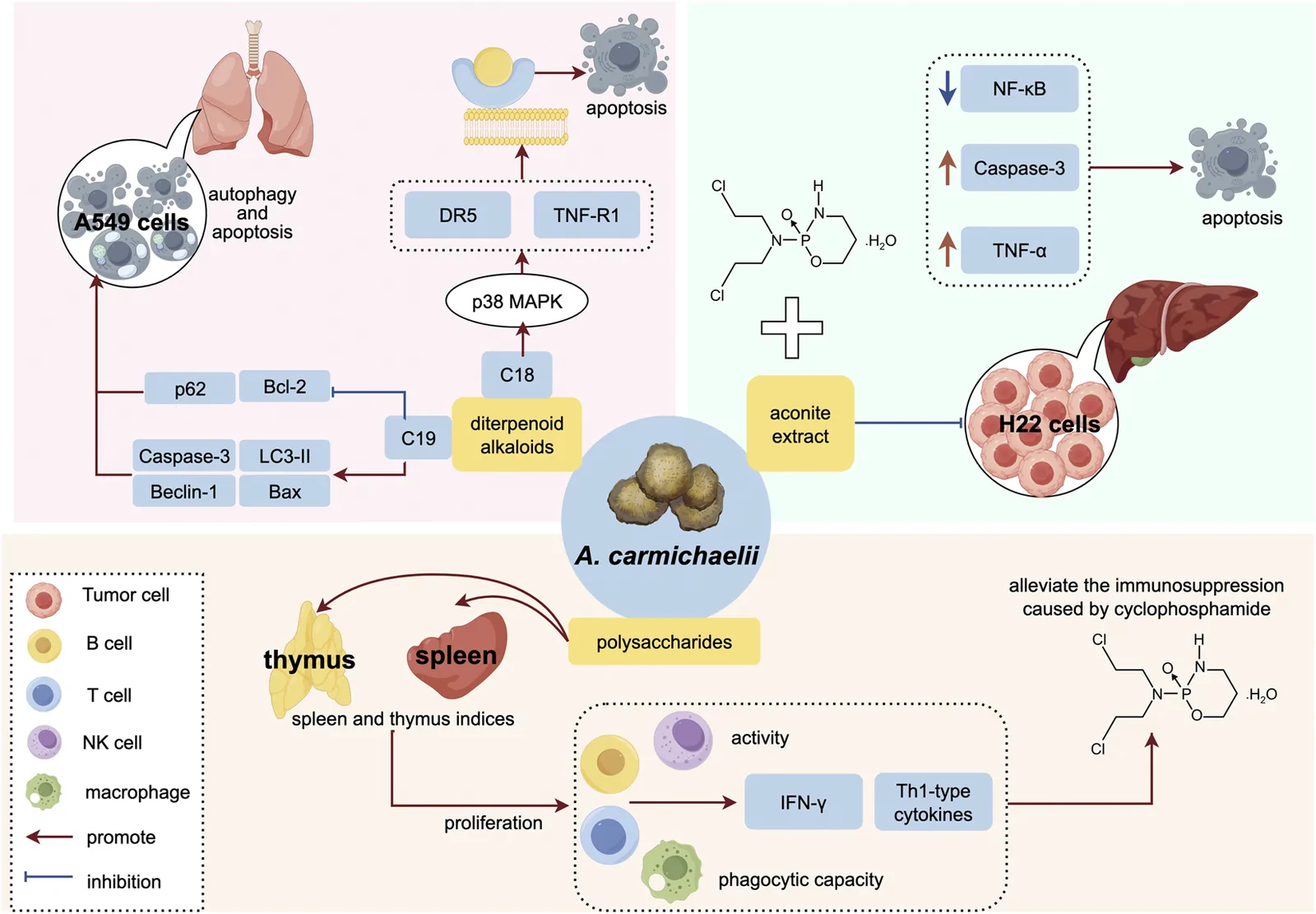
Pharmacological evidence for *A. carmichaelii*.

In YYFZBJS, *A. carmichaelii* is used as the processed product rather than raw aconite. Diester diterpenoid alkaloids from raw aconite are well known for high cardiotoxicity, whereas traditional processing promotes detoxification (de-esterification to less toxic monoester/acidic derivatives), thereby reducing aconitine-related toxicity (Gao et al., 2022; Zhang et al., 2022). In the clinical regimen, 6 g/day of processed Fuzi/aconite is used in combination therapy; clinical reports generally describe it as tolerable with cardiovascular monitoring and dose adjustment, while batch variability still warrants caution (Lin et al., 2023; Nie, 2020) (Pharmacological evidence for *A. carmichaelii* is shown in Figure 2).

### 3.3 P. scabiosifolia

Initially documented in *the Classic of the Divine Husbandman’s Materia Medica*, it is the whole grass of *Patrinia scabiosifolia* or *Patrinia villosa* of *Caprifoliaceae* family.

In modern pharmacological studies, *Patrinia villosa* has anti-cancer, anti-inflammatory, antioxidant, and liver-protective effects (Wang et al., 2022). Nearly four hundred chemical constituents have been isolated from *Patrinia villosa*, including phenylpropanoids, flavonoids, saponins, cyclic enol ether terpenes, sesquiterpenes, triterpenes, steroids, and volatile oils (Fan et al., 2022), of which flavonoids and saponins are the main antitumor components.

**FIGURE 3:**
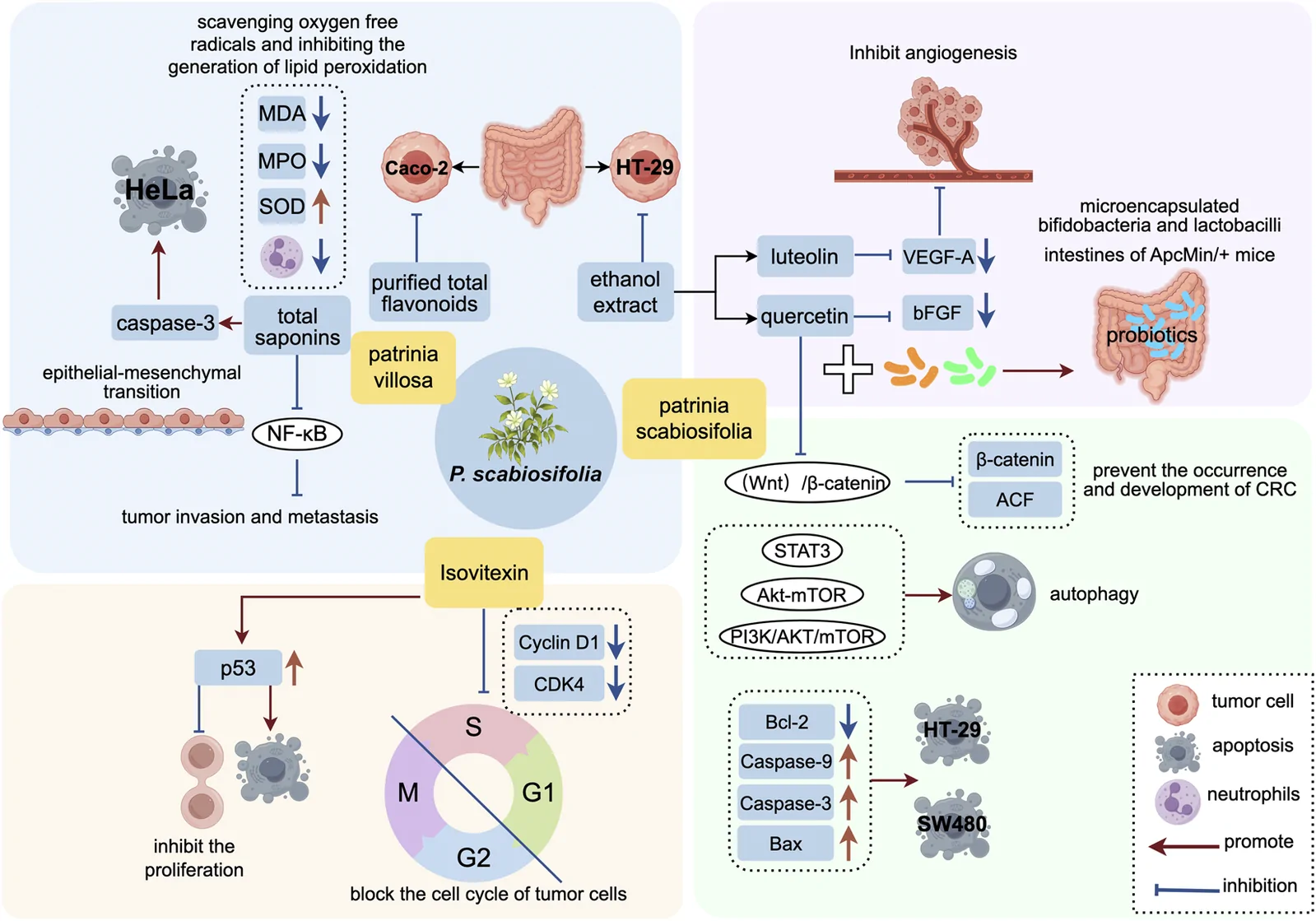
Pharmacological evidence for *P. scabiosifolia*.

It has been found that the purified total flavonoids of *Patrinia villosa* exhibit significant and specific inhibitory effects on intestinal cancer cells Caco-2 (Zhou et al., 2021). Total saponins of *Patrinia villosa* induce apoptosis in HeLa cells by activating the key enzyme of apoptosis, caspase-3 (Bao et al., 2017), and suppress tumor invasion and metastasis by inhibiting the NF-κB signaling pathway and epithelial–mesenchymal transition (EMT) (Xia et al., 2018). Aberrant activation of NF-κB signaling is a central driver of malignant progression across diverse tumor types; for example, WDR54 interacts with RBBP5 to enhance p65 transcription, thereby activating NF-κB signaling and promoting the proliferation and metastasis of hepatocellular carcinoma (Zhai et al., 2025). The convergence of these flavonoid and saponin components on NF-κB–dependent proliferation and EMT therefore provides a mechanistically coherent rationale for targeting this pathway, although the present evidence is largely derived from independent *in vitro* observations and warrants further orthogonal validation (Zhai et al., 2025; Quan et al., 2021). Consistent with this, it was found that the ethanol extract of *Patrinia scabiosifolia* inhibited the growth of HT-29 intestinal cancer cells as well as tumor angiogenesis, exerting anti-CRC effects (Chen et al., 2022). Further mechanistic studies revealed that *Patrinia scabiosifolia* induces HT-29 cell apoptosis by down-regulating the expression of mitochondrial pathway-associated protein Bcl-2, up-regulating the expression of Bax, and promoting the activation of downstream apoptosis-associated proteins Caspase-3 and Caspase-9 (Liu et al., 2013). Huang et al. (2013) also confirmed that the ethanol extract of *Patrinia scabiosifolia* could inhibit proliferation and alter the cell morphology of Caco-2 human colon cancer cells and reverses the chemoresistance of CRC cells, and it was positively correlated with the concentration (Huang et al., 2019). An animal model of transplanted tumors in nude mice further verified that *Patrinia scabiosifolia* intervention reduced tumor volume and inhibited tumor cell proliferation. Additionally, *Patrinia scabiosifolia* inhibits angiogenesis by suppressing the expression of HT-29 cell cycle proteins Cyclin D1 and CDK4, blocking the cells in the G1/S phase, and also inhibiting the expression of vascular endothelial growth factor VEGF-A, thus exerting an anti-CRC effect (Zhang et al., 2015). Previously, we analyzed the network-based pharmacology in conjunction with the GEO database, and confirmed that *P. scabiosifolia* and its active ingredient Isovitexin could inhibit CRC proliferation, induce apoptosis, and block the cell cycle through p53 by UPLC-MS sequencing and *in vivo* and *in vitro* experiments (Li J. et al., 2023). In clinical practice, *P. scabiosifolia* is commonly used in the treatments of diseases in digestive system, genitourinary system, dermatology, as well as oncology (Yang H. et al., 2023) (Pharmacological evidence for *P. scabiosifolia* is shown in Figure 3).

Overall, in YYFZBJS, the three Chinese Herbal Medicines, *C. lacryma, A. carmichaelii, and P. scabiosifolia*, have some active substances that induce apoptosis, regulate autophagy, and inhibit tumor cell proliferation through various mechanisms. These substances achieve the goals of anti-tumor effects and improving clinical symptoms, and have been proven effective in the treatment of various types of cancer (Table 1).

**TABLE 1:** Pharmacological evidence for active compounds of three constituent herbs in YYFZBJS.

TCM drugs	Original plants	Medicinal parts	Authenticated metabolites and mechanisms of action		Ref
*C. lacryma*	*Coix lacryma*	dried mature seed kernels	Coixenolide	Colorectal cancer: inhibits TNF-α-mediated epithelial mesenchymal transition (EMT) in CRC cells via NF-κB downregulation	Shi et al. (2018)
			Linoleic acid	CRC: reduces immune damage caused by 5-FU chemotherapy	Wang et al. (2024a)
			Coixol	CRC: inhibits the NF-κB and MAPK pathways	Lee et al. (2023)
			*C. lacryma* polysaccharide	Lung cancer:downregulates the gene and protein expression levels of S100A4 and interact with the S100A4-NMIIA binding site to inhibit the migration and invasion of A549 cells	Luo et al. (2017)
			*C. lacryma* oil	CRC: induce apoptosis	Ni et al. (2021)
*A. carmichaelii*	*Aconitum carmichaelii Debeaux*	a processed product of the root	Aconite C19 diterpenoid alkaloid	Lung cancer: induces autophagy in A549 cells via Beclin-1 upregulation	Zhang et al. (2022)
			Aconite C18 diterpenoid alkaloid	Multiple cancers:induces apoptosis	
			Aconite extract	Hepatocellular carcinoma:induces apoptosis in CRC cells via Caspase-3 activation	Ren and Zeng (2008)
			Aconite polysaccharides	Multiple cancers:alleviate the immunosuppression caused by cyclophosphamide	Fu et al. (2018)
*P. scabiosifolia*	*Patrinia scabiosifolia* or *Patrinia villosa*	whole plant	Purified total flavonoids in *Patrinia villosa*	CRC: inhibitory effects on intestinal cancer cells Caco-2	Zhou et al. (2021)
			Total saponins in *Patrinia villosa*	CRC: inducing apoptosis and inhibiting tumor invasion and metastasis	Xia et al. (2018)
			Ethanol extract of *Patrinia scabiosifolia*	CRC: inhibited the growth of HT-29 intestinal cancer cells and tumor angiogenesis, inhibit the proliferation and change the cell morphology of Caco-2 human colon cancer cells and reverse the chemoresistance of CRCcells	Chen et al. (2022), Liu et al. (2013)
			Isovitexin	CRC: inhibit CRCproliferation, induce apoptosis, and block the cell cycle through p53	Li et al. (2023a)

## 4 Study on the mechanism of action of YYFZBJS in the prevention and treatment of CRC

Although large-scale clinical data remain limited, existing evidence still supports its efficacy. The mechanisms described in this section are primarily based on *in vitro* cell and animal models, and are subject to limitations such as species differences (e.g., mouse gut microbiota differs from human microbiota) and dose discrepancies (animal study doses exceed commonly used clinical doses). Therefore, rigorous clinical validation is essential before therapeutic application.

### 4.1 Regulation of inflammation-cancer transformation

Chronic inflammation is one of the important mechanisms leading to the occurrence and development of tumors, accounting for approximately 25% of cancers (Chen et al., 2021). Inflammation-cancer transformation is a process of malignant cellular transformation that occurs when a large number of inflammatory cytokines infiltrate in the tissue microenvironment under the prolonged presence of chronic inflammation, leading to abnormal gene regulation and thus resulting in malignant cellular transformation. The distinction between inflammation-cancer transformation and chronic inflammation-induced carcinogenesis lies in the fact that the former emphasizes the dynamic process of cellular malignant transformation, while the latter highlights inflammation as a precipitating factor in carcinogenesis. In the process of inflammation-cancer transformation, a variety of inflammatory factors play an important role, including TNF-α, COX-2, IL-1β, IL-6, IL-8, and TGF-β1 (Li et al., 2022a). Among these factors, tumor necrosis factor TNF-α plays a significant role in the development of CRC and its associated malignant stroma, and is involved in almost all processes of tumorigenesis and development, including cell transformation, survival, proliferation, invasion, and metastasis (Kasprzak, 2021; Huang B. et al., 2021).

Studies have shown that TNF-α promotes the expression of β-catenin, c-myc, and cyclin D1 proteins in colon cancer cells and promotes the proliferation of colon cancer cells through the Wnt/β-catenin signaling pathway (Yu et al., 2018). Interleukins such as IL-1β, IL-6, and IL-8 have strong pro-inflammatory activity and also play significant roles in CRC.IL-1β mainly induces the occurrence of CRC by promoting the proliferation of colon cancer cells and altering the tumor microenvironment (Pastille et al., 2019; Bergman et al., 2013). IL-6 facilitates a microenvironment conducive to cancer cell metastasis by promoting cell mitosis, proliferation, metastasis, migration, and angiogenesis (Li J. et al., 2020).

An increasing number of studies indicate that ulcerative colitis (UC) is closely associated with the development of CRC, including a cohort study involving over 90,000 participants: 1,336 incident CRCs occurred in the UC cohort (1.29 per 1,000 person-years) versus 9,544 in matched reference individuals (0.82 per 1,000 person-years; HR 1.66, 95% CI 1.57–1.76). CRC deaths were also higher in UC (639, 0.55 per 1,000 person-years) compared with references (4,451, 0.38 per 1,000 person-years; HR 1.59, 95% CI 1.46–1.72). These data support the role of the chronic inflammatory microenvironment in driving inflammatory–cancer transformation in CRC (Olen et al., 2020). In this process, the inflammatory response activates a series of signal transduction pathways that affect cell growth, differentiation, and survival, ultimately leading to tumorigenesis. Therefore, the control of chronic inflammation, with the consequent reduction of inflammatory factor production at colorectal sites and the inhibition of inflammation-cancer transformation signaling, represents a promising research direction for tumor prevention and treatment. In the experiment of applying YYFZBJS to treat UC, it was found that the expression of IL-1β(71), TNF-α, IL-6,and IL-8 (Zhang et al., 2019) levels in the serum of patients with UC was significantly reduced. YYFZBJS, together with its active constituents isovitexin and quercetin, may help prevent CRC by suppressing NF-κB signaling, reducing pro-inflammatory cytokines such as IL-1β, IL-6, and IL-17, and increasing anti-inflammatory mediators including IL-10, IL-2, and IFN-γ (Yang L. et al., 2023; Mu et al., 2023; Hsieh et al., 2025). In addition, YYFZBJS inhibits TNF-α-induced β-catenin nuclear translocation by reducing NF-κB p65 phosphorylation, thereby interrupting the inflammatory signaling cascade that promotes malignant transformation (Zhang et al., 2025a). The compound sterol, as an effective active ingredient shared by the three herbs in YYFZBJS, has also been shown to promote the formation of an anti-inflammatory microenvironment, thereby contributing to prevent tumorigenesis (Henderson et al., 2012). Quercetin, a chemical component from *P. scabiosifolia*, reduces crypt β-catenin accumulation (Suh et al., 2007; Saud et al., 2013), thereby decreasing abnormal crypt foci and effectively preventing CRC (Zhang P. et al., 2024).

### 4.2 Relief of oxidative stress

Oxidative stress refers to an imbalance between ROS generation and antioxidant defense. Excessive ROS not only amplifies inflammation but also damages the intestinal mucosal barrier, promotes bacterial translocation and lipid peroxidation, and contributes to intestinal disease progression (Lv et al., 2016; Acevedo-Leon et al., 2023; Liu M. et al., 2019). Accumulating experimental and clinical evidence indicates that ROS-driven oxidative stress is involved in CRC initiation and progression. Triggered by risk factors such as radiation, smoking, and alcohol consumption, excessive ROS can induce oxidative DNA damage and activate both mitochondrial and death receptor-mediated apoptotic pathways, thereby facilitating colorectal carcinogenesis (Carini et al., 2017; Lin et al., 2018).

YYFZBJS exerts anti-oxidative stress effects by up-regulating the expression of Nrf2 and its downstream antioxidant protein HO-1 (Fang et al., 2018). Additionally, YYFZBJS significantly reduces the activity of murine myeloperoxidase (MPO), increases superoxide dismutase (SOD), and reduces malondialdehyde (MDA) and other oxidative stress-related indices in a mouse model of UC (Fang et al., 2018; Zhang Y. et al., 2024). In an *in vitro* antioxidant assay, the *C. lacryma* polysaccharide CSP1 and CSP2 increased the H_2_O_2_-induced activity of SOD, catalase (CAT), and glutathione peroxidase (GSH-Px) in the normal hepatocytes of WRL-68 humans, which acted as anti-oxidative stress protection (Jin et al., 2023). Aconite polysaccharides can scavenge DPPH radicals, hydroxyl radicals, and has strong antioxidant activity (Wen et al., 2016; Piang-Siong et al., 2017). Furthermore, the total saponins of *Patriniae Herba* increase SOD levels in rat colonic lesion tissue, decrease MDA levels and MPO activity, reduce neutrophil infiltration, and thereby scavenge oxygen free radicals and inhibit lipid peroxidation (Wang et al., 2015). Clinical studies have also confirmed that YYFZBJS significantly inhibits serum levels of 8-OHdG, 3-NT, hs-CRP, TNF-α, and IL-6 in patients with arthritis, and reduces inflammatory factors and oxidative stress in the patients’ systemic environment (Xie et al., 2023a). *In vitro* and *in vivo* experiments have demonstrated that Isovitexin, an active ingredient in patriniae herba, regulates mitochondrial autophagy and restores mitochondrial function through the upregulation of SIRT3, which enhances antioxidant capacity (Fan et al., 2025).

### 4.3 Improvement of gut microbiota imbalance

The gut microbiota is a complex micro-ecosystem involved in many pathological processes, which may lead to diseases such as inflammatory bowel disease (IBD), cancer, diabetes, and tumors (de Vos et al., 2022). In recent years, the role of gut microbiota in CRC has attracted widespread attention, with increasing evidence suggesting that gut microbiota and its metabolites are related to the occurrence, development, and treatment of CRC (Wong and Yu, 2023; Sears and Garrett, 2014). The pathogenic bacteria associated with CRC mainly include *Fusobacterium nucleatum*, pks + *Escherichia coli*, and enterotoxigenic *Bacteroides* fragilis (ETBF) (Liang et al., 2023; Louis et al., 2014). Its carcinogenic mechanisms include activating β-catenin and Wnt signaling pathways, inhibiting the functions of immune cells such as NK cells and T cells, thereby mediating tumor immune evasion, and promoting the development of Th17 and the transcription of IL-17 through the activation of Stat3, mediating intestinal epithelial cell proliferation and tumor formation, etc (Wong and Yu, 2023; Janney et al., 2020).

Relevant studies indicate that after YYFZBJS intervention, the content of gut microbiota genera in the feces of APC^Min/+^ mice changes significantly, suggesting that YYFZBJS can regulate the composition of the gut microbiota:increase beneficial bacteria species such as *Bifidobacterium* and reduce harmful bacteria such as *Bacteroides*, norank_f_Erysipelotrichaceae (Sui et al., 2020), thereby facilitating the restoration of the intestinal epithelial mucosal barrier (Zhang et al., 2025b). Decisively, the same study demonstrated by faecal microbiota transplantation (FMT) that transferring the YYFZBJS-conditioned microbiota into recipient mice recapitulated the key therapeutic phenotype: the accumulation of CD4^+^CD25^+^Foxp3^+^ regulatory T (Treg) cells in intestinal and mesenteric lymph nodes was reduced (from approximately 15%–8%), the tumour microenvironment was remodelled, the efficacy of PD-1 blockade was enhanced, and β-catenin phosphorylation was suppressed (Sui et al., 2020; Pardoll, 2012). Because the anti-tumour phenotype could be transferred through the microbiota alone, these data indicate that the YYFZBJS-conditioned microbiota is sufficient to mediate at least part of the formula’s anti-CRC effect, rather than changing in parallel with it. Moreover, the active ingredient quercetin in YYFZBJS, combined with microencapsulated bifidobacteria and lactobacilli, can increase the abundance of probiotics such as bifidobacteria and lactobacilli in the intestines of Apc^Min/+^ mice, regulate the gut microbiota, inhibit the secretory glycoprotein (Wnt)/β-catenin signaling pathway (Benito et al., 2021), reduce ACF, and thereby prevent the occurrence and development of CRC. Concurrently, elevated levels of short-chain fatty acids (SCFAs), metabolic products of bifidobacteria, activate the GPR43 pathway and suppress the release of inflammatory factors to inhibit tumor proliferation (Han et al., 2025; Cai et al., 2024).

However, complementary antibiotic-depletion experiments—administering YYFZBJS to mice with a pre-cleared microbiota—have not yet been reported, so microbiota-independent mechanisms cannot currently be excluded. Future studies should combine antibiotic preconditioning, germ-free recolonisation and metagenomic profiling to define which microbial taxa are necessary, sufficient or merely correlative for the anti-CRC effects of YYFZBJS, and to clarify the contribution of microbial metabolites such as short-chain fatty acids to the observed immune remodelling.

### 4.4 Inhibition of CRC cell proliferation and cell-cycle progression

Tumor development is intrinsically associated with excessive cell proliferation. Mutations in certain genes in cells result in enhanced expression of oncogenes and decreased or disappeared expression of anti-oncogenes, leading to uncontrolled growth and division of normal cells into cancer cells. MiR-103a-3p in CRC cells targets tumor suppressor kinase (LATS) 2, a core molecule of the Hippo/YAP1 pathway, and human homologous recombinant protein (SAV) 1 to promote hypoxia-inducible factor (HIF) 1A, which enhances CRC cell proliferation, migration, and invasive ability (Sun et al., 2020).

*In vitro* experiments proved that YYFZBJS inhibits the viability and proliferation of CRC cells, thereby suppressing the development of CRC (Li et al., 2022b). Additionally, YYFZBJS inhibits the proliferation of hepatocellular carcinoma cell lines Hepa1-6, HepG-2 and SMMC-7721 with dose- and time-dependent inhibitory effects (Sun et al., 2019). Chai et al. (2021) demonstrated that YYFZBJS inhibited chronic inflammation development and malignant transformation of adenoma mediated by phosphorylation of STAT3-mediated M2 macrophage polarization, thus inhibit colon tumor cell proliferation and infiltration. One of its active compounds, kaempferol, exerts anticancer effects by regulating the colon cancer cell cycle, inhibiting proliferation, and activating cell surface apoptosis receptors to induce apoptosis (Lee et al., 2014). Luteolin and quercetin, the active components contained in *P. scabiosifolia*, inhibited cell proliferation in a dose-dependent manner in two human CRC-derived cell lines, HCT15 and CO115 (Xavier et al., 2009). Quercetin has been shown to reduce proliferation, induce apoptosis, cause cell cycle arrest, and inhibit mitotic processes by regulating the molecular pathways of cell cycle proteins, PI3K/Akt, and mitogen-activated protein kinase (MAPK) (Reyes-Farias and Carrasco-Pozo, 2019; Li X. C. et al., 2023). In other tumors, such as ovarian cancer, YYFZBJS was able to effectively inhibit the proliferation of SK-OV-3 cells *in vitro*, and suppressed tumor growth *in vivo* (Pan et al., 2022). The main anti-tumor components in Patrinia, polysaccharides, giganteaside D (GD), and Patrinia monoterpene iridoid ether esters (PMIEE), inhibit cancer cell proliferation in liver cancer, breast cancer, and cervical cancer cells by downregulating Bcl-2, CDC2, and cyclin B1, and upregulating Bax and caspase-3 (Gong L. et al., 2021).

### 4.5 Promotion of apoptosis

Apoptosis is regulated by various genes, with the Caspases family and the Bcl-2 family serving as important regulatory genes for apoptosis. Caspases are involved in cell growth, differentiation, cytokine maturation, and apoptosis. Among them, Caspase-3 functions as a key effector molecule in the apoptosis pathway and serves as the primary executor of apoptotic processes (Pourhassanali et al., 2016). The Bax gene,a member of the Bcl-2 family, acts as a pro-apoptotic gene, and its overexpression induces cell death (Kim et al., 2018). By inducing apoptosis in tumor cells, it can improve the burden of various tumors, including CRC, and increase survival.

*In vitro* studies have demonstrated that YYFZBJS intervention in HCT116 cells for 48 h resulted in a rise in apoptosis rate from 21.0% to 53.5%, as detected by flow cytometry. While Western blot analysis confirmed a 2.1-fold upregulation of pro-apoptotic protein Bax, a 3.1-fold increase in Caspase-9 expression, a 1.4-fold rise in Caspase-3, and a 0.4-fold downregulation of anti-apoptotic protein Bcl-2. (Wei et al., 2023), thereby inhibiting the development of CRC. In the formula, *Patrinia herba* also induces Caspase-3 activation, promotes Bax expression, which subsequently leads to apoptosis in SW480 cells, resulting in inhibition of CRC cell proliferation and manifestation of anti-tumor effects (Huang F. et al., 2021). In parallel, these *Patriniae Herba* constituents have been shown to trigger apoptosis in liver, breast, and cervical cancer cells, as evidenced by decreased Bcl-2, CDC2, and cyclin B1 expression and increased Bax and caspase-3 levels (Gong L. et al., 2021). Our previous research results indicate that YYFZBJS inhibits the CDK/PI3K/Akt signaling pathway, induce G2/M phase cell apoptosis and cell cycle arrest (Li et al., 2022b), and inhibit the occurrence and progression of CRC. Furthermore, *C. lacryma* extract and *C. lacryma* oil have inhibitory effects on the growth and promote apoptosis in colon cancer HT-29 cells (Zhai et al., 2024). Additionally, Kanglaite injection promotes apoptosis in multiple tumor cell types by influencing relevant genes (Ma et al., 2024).

### 4.6 Regulation of cellular autophagy

Autophagy is involved in multiple physiological and pathological processes and plays a dual role in tumorigenesis. Key regulators, including mTOR, Beclin-1, p53, Akt, ERK, NF-κB, and ROS, are closely linked to cancer progression and treatment (Wang et al., 2020; White, 2016; Wang et al., 2018). Among the major regulatory pathways, PI3K/Akt is one of the best characterized; its activation promotes tumor growth partly by suppressing autophagy. Accordingly, inhibition of the PI3K/Akt pathway can induce autophagy and has emerged as a potential therapeutic strategy in cancer, as supported by the antitumor effects of PI3K, Akt, and mTOR inhibitors (Xie et al., 2021).

It was found that *in vitro* cellular experiments, YYFZBJS significantly downregulated the expression level of P62 protein in colon cancer HT-29 cells, and also inhibited the expression of p-PI3K and p-Akt, suggesting that YYFZBJS inhibits CRC progression by affecting cellular autophagy (Huang, 2023). Additionally, Kanglaite Injection could significantly upregulate the mRNA level of autophagy-related genes Beclin-1 and LC3 as well as the protein expression level, and promote autophagy in human hepatocellular carcinoma cells SMMC7721 (Gong S. et al., 2021). Quercetin, an active ingredient in *Patriniae herba*, induces apoptosis and autophagy in primary exudative lymphoma cells via inhibition of PI3K/Akt/mTOR and STAT3 signaling pathways (Granato et al., 2017). It also induces protective autophagy in gastric and breast cancer cells by inactivating the Akt-mTOR pathway (Jia et al., 2018; Wang et al., 2011).

### 4.7 Antitumor angiogenesis

Tumor occurrence and development depend on tumor angiogenesis, which is closely related to vascular endothelial cells and a variety of related factors that regulate angiogenesis (Roudsari and West, 2016). More and more studies have found that Chinese Herbal Medicine can inhibit tumor angiogenesis and affect the development of CRC by inhibiting the growth and migration of vascular endothelial cells, as well as regulating pro-angiogenic and inhibitory angiogenic factors (Li, 2014).

**FIGURE 4:**
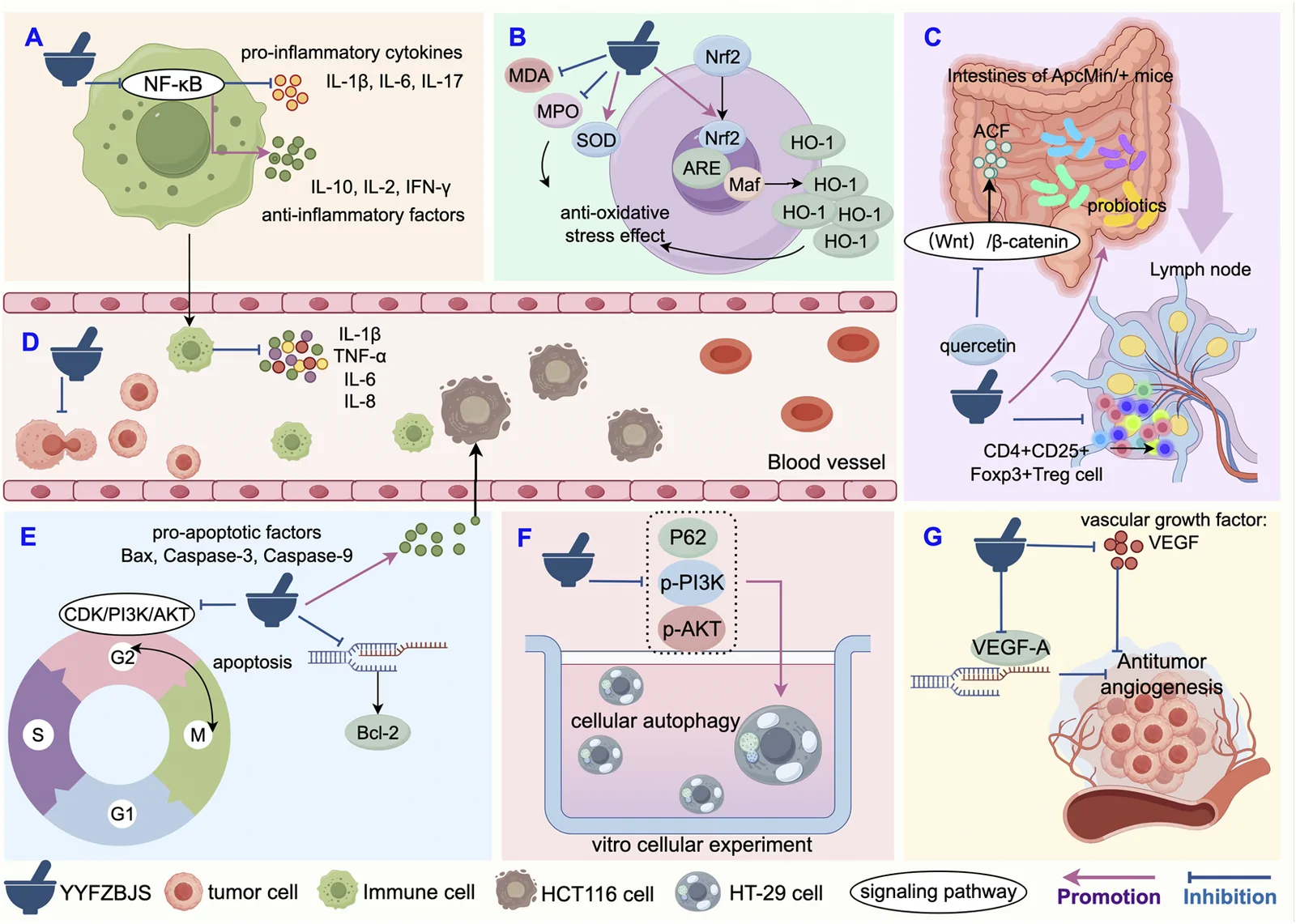
Study on the mechanism of action of YYFZBJS in the prevention and treatment of CRC. **(A)** Regulation of inflammation-cancer transformation. **(B)** Relief of oxidative stress. **(C)** Improvement of gut microbiota imbalance. **(D)** Inhibition of proliferation. **(E)** Promotion of apoptosis. **(F)** Regulation of cellular autophagy. **(G)** Antitumor angiogenesis.

Studies have proved that Kanglaite Injection can significantly inhibit neoangiogenesis and accelerate the entry of blood vessels into the decline phase, thus exerting anti-tumor effects (Han S. et al., 2024). Vascular endothelial growth factor (VEGF) is the most active pro-angiogenic factor, and the ethanol extract of *Patrinia scabiosifolia* significantly reduced the expression levels of VEGF-A mRNA and protein in CRC tumor tissues and human colon cancer HT-29 cells (Chen et al., 2013), inhibiting CRC development. The active ingredient quercetin is able to inhibit the activity of vascular growth factors VEGF and bFGF, consequently preventing tumor angiogenesis (Gao and Li, 2024). Luteolin, conversely, inhibits tumorigenesis and growth through a variety of pathways, including inhibition of VEGF and related signaling pathways (Li et al., 2019; Pratheeshkumar et al., 2012) and inhibition of angiogenesis molecules (Me et al., 2017) (Study on the mechanism of action of YYFZBJS in the prevention and treatment of CRC is shown in Figure 4).

Although available evidence suggests that YYFZBJS may regulate inflammation, oxidative stress, gut microbiota, proliferation, apoptosis, autophagy, and angiogenesis in CRC-related models, these findings are derived from multiple independent study systems, including cell experiments, animal models, clinical observations, and network pharmacology analyses. Therefore, the mechanisms summarized in this section should be regarded as an integrative and hypothesis-generating framework rather than a single experimentally validated pathway cascade. In particular, whether NF-κB or PI3K/Akt signaling is necessary for YYFZBJS-mediated suppression of cytokine production, proliferation, or tumor progression remains to be clarified by perturbation experiments using pathway inhibitors, activators, or genetic manipulation.

## 5 Clinical trial study on the treatment of CRC with YYFZBJS

Despite the increasing emergence of anti-tumor drugs and relatively comprehensive clinical treatment protocols for CRC, most of them only play the role of controlling the development of the disease, and it is difficult to play the role of curing the disease at root, while the morbidity and mortality of CRC are still showing a trend of increasing year by year. With the deepening of research, the application of YYFZBJS in the prevention and treatment of CRC is becoming more and more widespread.

In clinical trials, YYFZBJS Decoction uses 15 g of *C. lacryma* from Anhui, 6 g of *aconite* from Sichuan, and 12 g of *Patriniae herba* from Qinghai. It follows a standardized decoction process: soak in 500 ml of water for 30 min, bring to a boil over high heat, then simmer over low heat for 20 min. YYFZBJS combined with XELOX (oxaliplatin + capecitabine) regimen is safe, reliable and efficacious in the treatment of CRC, which can enhance immune function (post-treatment CD4+/CD8+ levels increased to 1.2 times baseline), modulate gut microbiota (elevated counts of *Lactobacillus*, *Enterococcus faecalis*, and *Bifidobacterium*), and reduce tumor marker expression (CEA and C199 decreased by 8.5 ng/ml and 21.9 U/ml respectively) (Lin et al., 2023). At the same time, modifications to the formula of YYFZBJS combined with XELOX regimen for the treatment of stage IV CRC (Cold-and-Heat-Staggering Type) significantly ameliorate the patients’ Chinese medicine symptoms, including abdominal distension, abdominal pain, and constipation, reduce the level of tumor markers CEA, CA199, and attenuate adverse effects of post-chemotherapy, and improve the patient’s tolerance to chemotherapy as well as overall quality of life (Nie, 2020). Injectable *C. lacryma* oil combined with FOLFOX4 in the treatment of postoperative patients with intestinal cancer can prolong progression-free survival from 88.14% to 81.36%, and improve patients’ immune resulting in increased serum levels of CD3^+^, CD4^+^, CD4+/CD8+, IgG, IgA, IgM, decreased levels of CEA, CA125, and CCSA-3, function and quality of life and attenuate the adverse effects of chemotherapeutic drugs, and reduced the level of relevant tumor markers (Qu et al., 2017) (Table 2).

**TABLE 2:** YYFZBJS clinical research summary.

Study design	Sample size	Intervention regimen	Primary outcome	Ref
Randomized controlled, single-center	n = 106	YYFZBJS + mesalazine, Oral, 4 weeks	Inflammatory factor levels decreased, IBDQ scores increased, beneficial bacteria *Bifidobacterium* and *Lactobacillus* counts rose, while harmful bacteria *Escherichia coli* and *Enterococcus* counts significantly decreased	Zhang et al. (2019)
Randomized controlled, single-center	n = 80	YYFZBJS, Enema, 4 weeks	Increased SOD and decreased TNF-α, IL-1β levels	Hu et al. (2020)
Randomized controlled, single-center	n = 105	YYFZBJS with Added Ingredients, Oral, 2 weeks	Alleviate oxidative stress and reduce levels of inflammatory factors	Xie et al. (2023 b)
Randomized controlled, single-center	n = 76	YYFZBJS + XELOX, Oral and injection, 6 weeks	Reduce tumor marker levels, improve fecal occult blood test results, decrease the incidence of common adverse reactions such as leukopenia, and enhance patients’ tolerance to chemotherapy	Nie (2020)
Randomized controlled, single-center	n = 78	YYFZBJS + XELOX, Oral and injection, 6 weeks	Enhance immune function, regulate gut microbiota, and reduce tumor marker expression	Lin et al. (2023)

## 6 Bioactive components and network-pharmacology-derived molecular targets of YYFZBJS in CRC

The treatment of diseases by traditional Chinese medicine is characterized by multi-components, multi-targets and multi-pathways. Based on the research and analysis of network pharmacology, the key active compounds in YYFZBJS can be analyzed for the treatment of CRC. Previously, we have analyzed the active compounds that contain the most target genes in YYFZBJS through network pharmacology, which are Isovitexin, luteolin, quercetin, kaempferol, sterol, and stigmasterol, etc. We hypothesized that these active compounds contribute to the inhibition of CRC by YYFZBJS and identified interactions between YYFZBJS active compounds and potential target genes related to CRC at the molecular level using molecular docking (Li et al., 2022b). In addition, to ensure these prioritized constituents can be reliably characterized and quantified across batches to support reproducible mechanistic interpretation. In the published literature, this is most commonly achieved by HPLC and UPLC-MS/MS for the flavonoid markers (isovitexin, quercetin, luteolin, kaempferol) from *P. scabiosifolia* and for the aconitine-type and benzoyl monoester diterpenoid alkaloids from *A. carmichaelii*; and by chromatographic fingerprinting combined with similarity analysis for whole-formula batch comparability, in accordance with the requirements of the Chinese Pharmacopoeia (2020 edition) (Chinese Pharmacopoeia Commission, 2020).

Isovitexin is recognized as an inhibitor of various tumor cells, including colon, breast, ovarian, prostate, esophageal, and pancreatic cancers (Ghanbari-Movahed et al., 2023). Isovitexin induces apoptosis and arrests the cell cycle of tumor cells through the p53 pathway (Li J. et al., 2023), as well as regulate the stemness of tumor cells, which further highlights its potential value in targeting tumor stem cells and inhibiting tumor progression (Ghanbari-Movahed et al., 2023). Isovitexin significantly reduces the proportion of CD133^+^ cells (from 49% to 10%) and inhibits the sphere and colony formation capabilities of tumor stem cells (Cao et al., 2019). Luteolin is a non-toxic flavonoid extracted from the leaves and stems of Luteolin (Wei et al., 2019). It has been found that luteolin inhibits cell cycle and induces apoptosis by affecting the metabolism of tumor cells, which leads to the inhibition of tumor cell growth (Selvendiran et al., 2006). In addition, luteolin inhibits the expression of MMP-9 and downregulate its enzymatic activity in HCT116 cells through the PI3K/Akt pathway (Wei et al., 2019). Quercetin is a flavonoid with multiple biological activities that inhibits a variety of enzyme activities and possesses antioxidant, anti-inflammatory, antifibrotic and antiviral activities (Maugeri et al., 2023). Relevant studies have revealed that quercetin exerts antitumor effects by targeting FAM198B, regulating the polarization of tumor-associated macrophages, and inhibiting macrophage secretion of tumor-promoting related cytokines, which subsequently inhibits CRC progression (Deng et al., 2025). Kaempferol also belongs to the flavonoids group, which has the effects of cancer inhibition, anti-infection, anti-inflammation, and protection of intestinal barrier function (Chen J. et al., 2023). *In vitro* experiments demonstrated that kaempferol reversed chemoresistance to 5-FU in CRC cells by inhibiting PKM2-mediated glycolysis (Wu et al., 2022). In addition, kaempferol reduces the expression level of CRC suppressor DACT2 gene and inhibits its downstream Wnt/β-catenin pathway by regulating the protein expression of DNMTs and HDACs, which arrests the tumor cell cycle, induces apoptosis, and inhibits migration (Lu et al., 2018). Concurrently, kaempferol may reduce the body’s inflammation through the NF-κB inflammatory signaling pathway and enhance the body’s antioxidant capacity (Choi et al., 2018).

In summary, the key active components in YYFZBJS—including coixenolide (*C. lacryma*), aconitine (*A. carmichaelii*), isovitexin (*P. scabiosifolia*), luteolin (*P. scabiosifolia*), quercetin (*C. lacryma*), and kaempferol (*A. carmichaelii*)—exert anti-CRC effects through complementary mechanisms such as promoting apoptosis, suppressing proliferation and metastasis, modulating inflammation and oxidative stress, and enhancing chemosensitivity via pathways including NF-κB, PI3K/Akt/mTOR, p53, and PKM2-mediated glycolysis. These findings highlight the multi-component and multi-target characteristics of YYFZBJS and support its potential as an adjuvant strategy for CRC treatment (Zhang et al., 2022; Li J. et al., 2023; Yang L. et al., 2023; Li et al., 2022b; Wu et al., 2022; Wang T. et al., 2024) (Tables 3, 4).

**TABLE 3:** Key active compounds of YYFZBJS.

Components	Pathways or targets	Effect	Ref
Isovitexin (*P. scabiosifolia*)	p53	Induce apoptosis and block the cell cycle of tumor cells	Li et al. (2023a)
Luteolin (*P. scabiosifolia*)	PI3K/Akt	Inhibit the expression of MMP-9 and downregulate its enzymatic activity in HCT116 cells	Wei et al. (2019)
Quercetin (*P. scabiosifolia*)	FAM198B	Regulating the polarization of tumor-associated macrophages, and inhibiting macrophage secretion of tumor-promoting related cytokines	Zheng (2023)
Kaempferol (*A. carmichaelii*)	PKM2	Reversed chemoresistance to 5-FU in CRC cells	Wu et al. (2022)
	DNMTs and HDACs Wnt/β-catenin	Arrests the tumor cell cycle, induces apoptosis, and inhibits migration	Lu et al. (2018)
	NF-κB	Reduce the body’s inflammation and enhance the body’s antioxidant capacity	Choi et al. (2018)

**TABLE 4:** YYFZBJS summary of dosage forms, dosages, and treatment courses for clinical and preclinical studies.

Research type	Intervention method	Dosages	Treatment courses	Effect
clinical studies	YYFZBJS oral	one dose daily (15 g coix seed, 6 g aconite, 12 g patrinia root)boiled in 500 mL water and divided into two warm doses	4–8 weeks	warm Yang and fortify the spleen, clear heat, and resolve dampness, drain pus and remove toxins, break up blood stasis and disperse knots
animal studies	YYFZBJS gavage	20 g/kg/d	6 weeks	regulate gut microbiota
*in vitro* studies	YYFZBJS-containing serum acts on HT-29 cells	10%–20%	48 h	inhibit proliferation

The above flavonoids (isovitexin, luteolin, etc.) are ubiquitous in plants, but their synergistic effects with other compounds in YYFZBJS (e.g., coixenolide, aconitine) and specific targeting of CRC-related pathways (e.g., p53, PI3K/Akt) distinguish YYFZBJS, from single-compound interventions (Li J. et al., 2023; Li et al., 2022b).

## 7 Discussion

The MAPK cascade engaged by *A. carmichaelii* alkaloids is far from monolithic. In other tumour types, MAPK signalling stratifies into functionally distinct subgroups with divergent prognostic and therapeutic implications, as illustrated by molecular-subtype analyses of MAPK-driven gliomas. The immune-modulatory effects of YYFZBJS may likewise converge on emerging targets that reshape antigen presentation and effector-cell recruitment in the tumour microenvironment; in particular, polarization of tumour-associated macrophages toward an immunosuppressive M2 phenotype—recently shown in colon cancer to be driven by Galectin-9 — may be a key node at which YYFZBJS-derived flavonoids exert their cytokine-modulating effects. Future mechanistic studies should couple direct cytotoxicity assays with TAM-polarization read-outs and antigen-specific T-cell function to position YYFZBJS within this emerging immune landscape.

## 8 Summary, limitations and future directions

### 8.1 Principal mechanisms

YYFZBJS exerts anti-CRC activity through coordinated regulation of inflammation, oxidative stress, and the gut ecosystem. Mechanistically, the Nrf2/HO-1 axis serves as the central molecular target of this antioxidant arm: YYFZBJS upregulates Nrf2 and its downstream effector HO-1, thereby mitigating ROS-associated intestinal injury and limiting pathogenic bacterial colonization; in parallel, it reduces endotoxemia-driven inflammatory signaling, characterized by decreased pro-inflammatory cytokine levels such as IL-6. Consistent with this antioxidant effect, YYFZBJS reshapes the gut microbiota (e.g., increases *Bifidobacterium*), enhances SCFA production, and further suppresses NF-κB activation, forming a self-reinforcing cycle of “antioxidation–microbiota regulation–inflammation suppression” (Fang et al., 2018; Sui et al., 2020; Zhang Y. et al., 2025).

As a classical multi-herb formula, YYFZBJS overcomes the limitations of single-herb approaches via multi-component synergy across distinct functional nodes. Specifically, *C. lacryma* polysaccharides primarily promote beneficial microbiota capable of producing SCFAs, while polysaccharides from both *C. lacryma* and *A. carmichaelii* contribute to immune modulation. In addition, aconite alkaloids exert anti-inflammatory effects, whereas flavonoids from *P. scabiosifolia* mainly inhibit tumor cell cycle progression and promote apoptosis. Together, these coordinated actions help break the inflammation-driven vicious cycle in the intestinal microenvironment (Zhang Y. et al., 2024; Ge et al., 2024).

Beyond the inflammation–oxidative stress axis, NF-κB and the PI3K/Akt/mTOR cascade represent the two principal molecular targets of YYFZBJS in CRC, with NF-κB disruption providing the key mechanistic link to attenuation of the inflammation–cancer transformation pathway. NF-κB acts as both an inflammatory mediator and oncogenic transcription factor, driving multiple pro-inflammatory cytokines and establishing a self-perpetuating feedback loop that sustains chronic inflammation and promotes malignant transformation (Yang L. et al., 2023). YYFZBJS interrupts this pathological loop through convergent actions: total saponins from *P. scabiosifolia* and *C. lacryma* oil reduce NF-κB p65 expression and thereby lower serum pro-inflammatory cytokines while increasing anti-inflammatory mediators (Shi et al., 2018; Xia et al., 2018); meanwhile, isovitexin and quercetin further suppress inflammatory cytokine production and inhibit NF-κB pathway activation (Hsieh et al., 2025; Wang W. et al., 2025). In parallel, YYFZBJS targets the PI3K/Akt/mTOR axis, a central survival/proliferation signaling cascade; for example, quercetin inhibits proliferation, migration, invasion, and metastasis by modulating cyclin-related molecules and downstream PI3K/Akt/mTOR–MAPK signaling (Leiphrakpam and Are, 2024; Wu et al., 2025). Consistently, *C. lacryma* oil promotes apoptosis via the PI3K/Akt pathway by activating Caspase-3 and regulating Bax/Bcl-2 (Ni et al., 2021), and YYFZBJS as a whole triggers G2/M-phase arrest and apoptosis through inhibition of the CDK/PI3K/Akt axis (Li et al., 2022b).

### 8.2 Limitations

Nevertheless, several limitations remain. Mechanistic investigations of YYFZBJS in CRC are still largely confined to *in vitro* and animal models, with relatively few clinical studies. Existing clinical evidence is constrained by small sample sizes, single-center designs, and a lack of rigorously designed randomized controlled trials (RCTs), and the proposed mechanisms have yet to be fully validated in patients. Most clinical studies employ integrated Chinese and Western medicine regimens, which, although effective, complicate the independent evaluation of YYFZBJS within the TCM framework of holistic regulation and syndrome differentiation. Moreover, there is a paucity of long-term safety data (≥12 months) and comprehensive pharmacokinetic profiling (e.g., half-life, tissue distribution). To better guide clinical practice, future multicentre RCTs with sample sizes ≥300 should evaluate YYFZBJS as monotherapy or in combination with standard adjuvant therapy, with stratified randomization based on TNM stage and typical TCM syndromes such as spleen deficiency with dampness accumulation or damp-heat with blood stasis-toxin. Recommended primary endpoints include progression-free survival (PFS) and overall survival (OS), while secondary endpoints should assess tumor markers (CEA, CA19-9), TCM symptom scores (e.g., abdominal distension, pain) and quality of life (EORTC QLQ-C30). Parallel monitoring of hepatic and renal function, cardiovascular indices, and gut microbiota stability, combined with population pharmacokinetic modelling, will help to develop individualized dosing strategies for patients of different ages and body weights.

A further limitation concerns the heterogeneity of the preclinical evidence base itself. Most mechanistic claims summarized in Sections 3, 4 rely on a limited number of cell lines or single rodent models without genetic or pharmacological rescue experiments; observed changes in Bax/Bcl-2, p-PI3K/p-Akt, NF-κB p65 or LC3-II/p62 should therefore be regarded as correlative rather than causally validated, and network-pharmacology or molecular-docking predictions require orthogonal experimental confirmation before being read as evidence of species-level activity. Reporting of dose range, minimal effective concentration, treatment duration, and positive/negative controls is also uneven across cited studies, constraining direct cross-comparison. The mechanistic framework presented here should accordingly be regarded as an integrative working model whose nodes vary in evidence strength.

### 8.3 Future directions

It should also be emphasized that the signaling network proposed in Figure 4 is hypothetical and integrative. The current evidence supporting YYFZBJS-mediated regulation of inflammation, oxidative stress, gut microbiota, proliferation, apoptosis, autophagy, and angiogenesis comes from multiple independent experimental systems rather than from a single study that simultaneously validates all nodes of the network. For example, NF-κB-related inflammatory regulation, PI3K/Akt-mediated proliferation or autophagy, Nrf2/HO-1-mediated antioxidant responses, microbiota remodeling, and VEGF-associated angiogenesis suppression have generally been examined in separate models. Therefore, these pathways should be interpreted as potentially interconnected but not yet experimentally integrated. Future studies should combine YYFZBJS treatment with targeted perturbation of NF-κB or PI3K/Akt signaling, followed by downstream measurements such as IL-1β, IL-6, TNF-α, IL-10, p-p65/p65, p-PI3K/p-Akt, Ki-67, CCK-8/EdU proliferation assays, apoptosis rate, and VEGF-A expression. Demonstrating that pathway inhibition mimics, enhances, or abolishes the effects of YYFZBJS would provide stronger causal evidence for the proposed network model.

In parallel, given the limitations of existing studies and the paucity of high-quality clinical data, further work is required to elucidate its precise molecular mechanisms, clarify long-term safety and efficacy, and establish standardized quality-control systems. The modernization of YYFZBJS-based CRC therapy will depend on the development of safe, effective and scientifically validated preparations suitable for widespread clinical application. Moreover, comprehensive research specifically summarizing the effects of YYFZBJS on CRC through NF-κB, PI3K/Akt and related pathways remains scarce. This review therefore aims to expand current knowledge on the role of YYFZBJS in CRC management via these signaling networks and to highlight its substantial potential as a modern, evidence-based adjuvant therapy.

## Glossary

ACF: aberrant crypt foci
Akt: protein kinase B
Bax: BCL2-associated X protein
Bcl-2: B-cell lymphoma 2
BXPC3: Biopsy xenograft of Pancreatic Carcinoma line-3
CA: carbohydrate antigen125
CAFs: cancer-associated fibroblasts; CCSA-3, colon cancer-specific antigen-3
CDK: cyclin-dependent kinase
CEA: carcinoembryonic antigen
COX-2: cyclooxygenase-2
FOLFOX4: panitumumab plus oxaliplatin, fluorouracil, and leucovorin
Foxp3: forkhead box P3
DACT2: dishevelled binding antagonist of beta catenin 2
DNA: Deoxyribonucleic Acid
DNMTs: DNA methyltransferases
DR5: death receptor 5
DPPH: 2,2-Diphenyl-1-picrylhydrazyl; ECM, extracellular matrix
ERK: extracellular signal-regulated kinase
FGF: fibroblast growth factor
GEO: gene expression omnibus
HDACs: histone deacetylase
hs-CRP: hypersensitive C-reactive protein
IFN-γ: interferon-γ; IgG, immunoglobulin G
IL: interleukin; MAPK, mitogen-activated protein kinase
MiR: microRNA
MMP: matrix metalloproteinase
mTOR: mechanistic target of rapamycin
NF-κB: nuclear factor Kappa-light-chain-enhancer of activated B Cells
NK: natural killer cell
Nrf2: nuclear factor erythroid 2-related factor 2
NMIIA: Non-muscle myosin IIA
OGD/R: oxygen-glucose deprivation/reperfusion
PI3K: phosphoinositide 3-Kinase
PKM2: pyruvate kinase M2
p53: upregulated modulator of apoptosis
SIRT3: sirtuin 3
STAT3: Signal transducer and activator of transcription 3
TCM: traditional chinese medicine
TGF: transforming growth factor
TNF-a: tumor necrosis factor alpha
UPLC-MS: ultra-high performance liquid-chromatography
Treg: regulatory T cells
VEGF: vascular endothelial growth factor
TNFR: Tumor necrosis factor receptor
Wnt: wingless-related integration site.
YAP: yes-associated protein
8-OHdG: 8-oxo-2′-deoxyguanosine
3-NT: 3-nitro-l-tyrosine
