# Supplementary material for: Therapeutic potential and molecular mechanisms of Yi-Yi-Fu-Zi-Bai-Jiang-San in the treatment of colorectal cancer
Source: Front Pharmacol. 2026 Aug 14;17:1813778. doi: 10.3389/fphar.2026.1813778 (PMC13522946; doi:10.3389/fphar.2026.1813778)
Supplement: Supplementary file 2 [file Table1.docx]

**Literature search and study selection**

A structured literature search was conducted to ensure comprehensiveness and reproducibility of evidence synthesis. PubMed, Web of Science, Embase, Ovid MEDLINE, CNKI, and Wanfang Data were searched for studies published between January 2000 and February 2026, without initial restriction on study type. The following key terms were used in combination with Boolean operators: (“Yi-Yi-Fu-Zi-Bai-Jiang-San” OR “YYFZBJS”) AND (“colorectal cancer” OR “CRC”), supplemented by additional searches for individual herbal components (*Coix lacryma-jobi*, Aconitum *carmichaelii*, *Patrinia scabiosifolia*) and representative bioactive compounds (e.g., quercetin, luteolin, kaempferol, isovitexin). Reference lists of relevant articles were also manually screened to identify additional studies. Eligible studies included experimental (in vitro and in vivo), clinical, and mechanistic investigations related to CRC or cancer-associated biological processes, including inflammation, oxidative stress, gut microbiota regulation, proliferation, apoptosis, autophagy, and angiogenesis. After removal of duplicates, studies were screened by title/abstract and subsequently by full-text assessment according to predefined inclusion and exclusion criteria. Given the heterogeneity of study designs, a qualitative narrative synthesis was performed, and evidence was integrated according to major mechanistic themes rather than quantitatively pooled. This review followed a PRISMA-informed structured literature search and selection framework to enhance transparency and reproducibility, while being conducted as a narrative-integrative review rather than a fully registered systematic review.
